# An *in vivo *root hair assay for determining rates of apoptotic-like programmed cell death in plants

**DOI:** 10.1186/1746-4811-7-45

**Published:** 2011-12-13

**Authors:** Bridget V Hogg, Joanna Kacprzyk, Elizabeth M Molony, Conor O'Reilly, Thomas F Gallagher, Patrick Gallois, Paul F McCabe

**Affiliations:** 1 School of Biology and Environmental Science, University College Dublin, Dublin 4, Ireland; 2 School of Agriculture, Food Science and Veterinary Medicine, University College Dublin, Dublin 4, Ireland; 3 Faculty of Life Science, University of Manchester, Michael Smith Building, Oxford Road, Manchester, M13 9PT, UK; 4 Syngenta, Jealotts Hill International Research Centre, Bracknell, Berkshire, UK

**Keywords:** apoptotic-like, programmed cell death, Arabidopsis, root hair

## Abstract

In *Arabidopsis thaliana *we demonstrate that dying root hairs provide an easy and rapid *in vivo *model for the morphological identification of apoptotic-like programmed cell death (AL-PCD) in plants. The model described here is transferable between species, can be used to investigate rates of AL-PCD in response to various treatments and to identify modulation of AL-PCD rates in mutant/transgenic plant lines facilitating rapid screening of mutant populations in order to identify genes involved in AL-PCD regulation.

## Background

Programmed cell death (PCD) can be described as the organised destruction of a cell [1,2] and plays an important role in many plant developmental pathways including xylogenesis, embryogenesis, root and leaf development and senescence [3,4]. During the hypersensitive response it provides a defence response against invading pathogens [5] and has been shown to be part of the plants response to environmental stresses [6].

While there are several different forms of plant PCD [reviewed [7,8]] and differences between plant PCD and apoptosis, the type of PCD which we observe in cell suspension cultures, apoptotic-like PCD (AL-PCD), has some shared features with apoptosis [2]. For example, in response to biotic and abiotic stress, suspension culture cells have been shown to undergo nuclear condensation and internucleosomal DNA cleavage [6], activate caspase-like activity [9] and release cytochrome *c *from the mitochondria [10]. In addition, both animal and plant cells demonstrate condensation of the cytoplasm. In plant cells the retraction and condensation of the cytoplasm leaves a visible gap between the cell wall and the plasma membrane resulting in a specific corpse morphology [11], a hallmark feature that has been a useful tool in quantifying rates of AL-PCD in plant suspension cultures from a wide variety of species. For example, the effects of chemically induced cell death in sycamore cell cultures [12] and in soybean cells [13], the effect of HR elicitors on PCD rates in tobacco [14] and *Arabidopsis thaliana *[15] and the role of hydrogen peroxide on the induction of PCD in *Arabidopsis thaliana *[15], *Glycine max *[16] and *Nicotiana plumbaginifolia *[17] have all been investigated in cell suspension cultures. More recently, in transgenic lines, Burbridge *et al*. [18] showed that AL-PCD morphology could be used to investigate the effects of elevated levels of peroxidase on the AL-PCD induction threshold in transgenic cell suspension cultures of *Nicotiana tabacum*.

The AL-PCD associated corpse morphology has also been described in cells of whole plants. Delorme *et al*., [19] showed that senescing cucumber cotyledon cells exhibit retraction of the cytoplasm and this retraction was associated with internucleosomal cleavage. During the hypersensitive response cells surrounding the site of infection undergo PCD, with the retraction of the cytoplasm thought to be a method for controlling the spread of infection [5]. In fact, there are numerous examples in plants in which cytoplasm condensation occurs during PCD. These include, leaf morphogenesis in *Monstera *[20] and lace plant leaves [21], in the final stages of senescence [19,22] or in tissues undergoing the hypersensitive response [23,24]. The lace plant *Aponogeton madagascariensis *provides *in vivo *system for studying PCD in real time [25] however in other species the cells undergoing AL-PCD in whole plants are often buried within the tissue and large-scale scoring of individual cells to evaluate changes in cell death rates is not always feasible. While cell suspension cultures have provided a model to investigate AL-PCD in individual cells, they can be labour intensive to establish and time is required to achieve a mature stable cell line. As such they are not amenable to investigation of altered AL-PCD rates in mutant/transgenic lines in which large amounts of time have already been invested and many lines may need to be scored. There is a need therefore to develop an *in vivo *model which utilises the AL-PCD morphology to evaluate changes in rates of PCD in whole plants.

In order to provide a simple, rapid and *in vivo *model for the investigation of AL-PCD rates we assessed a range of cell types, for their suitability to facilitate large-scale scoring of AL-PCD in whole plants. An important criterion in assessing their suitability being that they exhibit the AL-PCD associated corpse morphology when subjected to a known inducer of AL-PCD such as heat, and that these cells are easily visualized in large numbers. Root hairs are single cells, readily observed by light microscopy and the response of each individual root hair subjected to specific treatments can be determined easily. Using the corpse morphology as a visual indicator of AL-PCD, they provide a quick *in vivo *model for quantifying rates of AL-PCD. In addition, many root hairs can be scored per plant and exogenous compounds can be added to the root system and their effect on AL-PCD rates investigated. Plants can be grown quickly and easily and results obtained in a matter of days. Root hairs can also be used to investigate AL-PCD rates in mutant/transgenic plant lines. Lines whose root hairs have an altered AL-PCD response can then be selected for the production of cell suspension cultures if so desired.

We describe in this paper a simple model for evaluating AL-PCD *in vivo*. Using *Arabidopsis thaliana *we show that results can be obtained (from seed to scoring) in 6 days and that induction of AL-PCD in root hairs can be achieved both by heat (abiotic stress) and chemical stress (ethanol, acetylsalicylic acid, hydrogen peroxide and sodium chloride treatment). The AL-PCD phenotype is comparable to that observed in cell suspension cultures and the response to heat stress almost identical. We also demonstrate that AL-PCD of *Arabidopsis thaliana *root hairs is suppressed by Ac-DEVD-CHO, a caspase-3 inhibitor. To test whether this system can be used to screen for lines with an altered AL-PCD response we present results from *At-DAD1 *(Defender Against Apoptotic Cell Death) over-expressor line of *Arabidopsis thaliana *DAD-1 [26].

## Results and Discussion

### AL-PCD morphology

**Figure 1:**
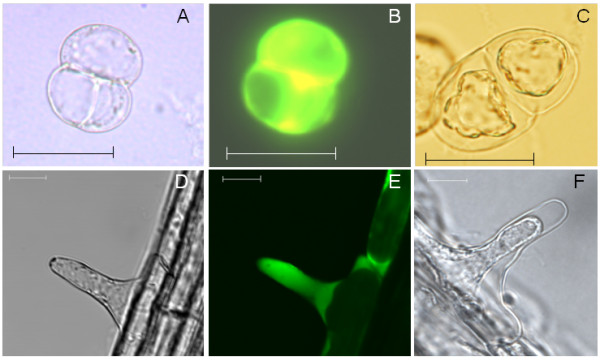
**AL-PCD morphology in cell suspension cultures and root hairs of *Arabidopsis thaliana***. **(A)**, **(B) **Living cells from a seven day old suspension culture stained with FDA and viewed under white **(A) **or fluorescent light **(B)**. **(C) **Two cells from a seven day cell suspension culture 24 hr after a 10 min heat shock at 55°C, showing retraction of the cytoplasm, a characteristic hallmark of AL-PCD. When stained with FDA these cells do not fluoresce indicating that they have undergone cell death. **(D)**, **(E) **Root hair stained with FDA and viewed under white **(D) **or fluorescent light **(E)**. Unlike root hairs treated with heat no retraction of the cytoplasm can be observed. **(F) **Root hair of *Arabidopsis thaliana *6 hr after a 10 min heat shock at 55°C. The root hair shows retraction of the cytoplasm and no fluorescence following FDA treatment indicating it has undergone AL-PCD. Scalebars: 40 μm A-C and 10 μm D-F.

Much progress has been made into understanding how AL-PCD occurs in plants through the use of suspension cultures. However there has been no readily available/widely applicable *in vivo *model of plant AL-PCD. To use root hairs as an *in vivo *system for the investigation of AL-PCD it was first established whether they exhibit morphological markers of AL-PCD when subjected to a PCD inducing stress. Heat shock has been shown to induce AL-PCD in *Arabidopsis thaliana *cell suspension cultures. McCabe and Leaver [11] showed that when *Arabidopsis thaliana *cells are subjected to a 10 min, 55°C heat shock over 90% of the cells die by AL-PCD, while the remaining cells died in an uncontrolled manner termed necrosis, a rapid death that is not associated with cytoplasmic retraction. Viable cells do not exhibit any protoplast retraction (Figure 1A) and fluoresce green when stained with the vital stain fluorescein diacetate (FDA) (Figure 1B). In contrast, cells that have undergone AL-PCD (Figure 1C) show marked protoplast retraction and, unlike living cells, do not fluoresce when incubated with FDA. Viable root hair cells (Figure 1D) also fluoresce when stained with FDA (Figure 1E). As with cultured cells, root hair cells that have undergone a 55°C heat shock display typical AL-PCD morphology. Condensation of the cytoplasm and retraction of the plasma membrane away from the cell wall towards the base of the root hair cell can clearly be seen in root hair cells of *Arabidopsis thaliana *6 hr after a ten minute heat shock at 55°C (Figure 1F). In addition, as observed with cell suspension cultures, root hair cells exhibiting this morphology do not fluoresce when stained with FDA at this timepoint (6 hr after heatshock). Although the severity of the condensation and retraction of the cytoplasm may vary from root hair to root hair (data not shown) this is a readily scorable phenotype. When used in conjunction with FDA staining, root hairs that have undergone AL-PCD are readily distinguishable from alive/necrotic root hair cells.

### Caspase inhibitor study

**Figure 2:**
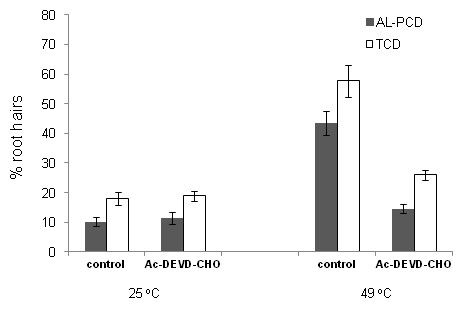
**The effect of caspase inhibitor on rates of heat-induced AL-PCD in *Arabidopsis thaliana***. Five day old seedlings of *Arabidopsis thaliana*, with or without, 1 hr preincubation in 1 μM Ac-DEVD-CHO (caspase-3 inhibitor) were subjected to a heat treatment at 49°C for 10 min or no heat treatment. Root hairs were scored 24 hr later. Root hairs deemed to have undergone cell death, determined by the lack of FDA staining, were scored for AL-PCD characterised by retraction and condensation of the cytoplasm. Those root hairs showing neither FDA staining nor retraction of the cytoplasm, were scored as necrotic. Rates of AL-PCD or Total Cell Death (TCD) (TCD = necrotic + AL-PCD) were plotted. Error bars = standard error of n = 3 replicates.

In seedlings of *Arabidopsis thaliana*, caspase-like activity can be induced in response to ultraviolet-C induced AL-PCD. This activity could be inhibited by pre-incubation with the caspase inhibitor Ac-DEVD-CHO [27]. In order to investigate a link between the AL-PCD observed in root hairs and caspase-like activity, the effect of preincubation of *Arabidopsis thaliana *seedlings with caspase-3 inhibitor, Ac-DEVD-CHO was examined. Pre-incubation with 1 μM Ac-DEVD-CHO significantly reduced the rates of AL-PCD induced by heat shock at 49°C from 43.5% to 14.6% (Figure 2) and had no effect on root hair viability in root hairs in which AL-PCD had not been induced. It seems, therefore, that caspase-like activity plays a role in the regulation of AL-PCD in root hairs.

### Response of root hairs to a range of temperatures

**Figure 3:**
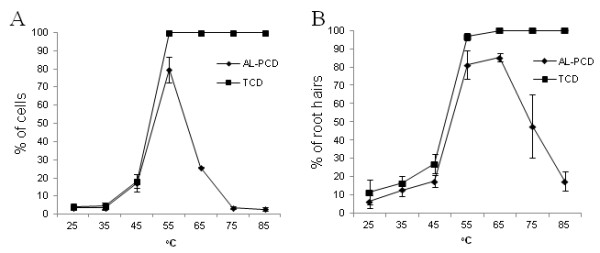
**The effect of heat treatment on rates of AL-PCD in *Arabidopsis thaliana***. Rates of AL-PCD were determined in **(A) **Seven day old cell suspension cultures subjected to a range of temperatures for 10 min and scored 24 hr later and **(B) **root hairs subjected to a range of temperatures for ten minutes and scored 6 hr later. Cells/root hairs showing retraction and condensation of the cytoplasm and no FDA staining were deemed to have undergone AL-PCD. Those root hairs/cells showing neither FDA staining nor retraction of the cytoplasm were scored as necrotic. Rates of AL-PCD or Total Cell Death (TCD) (TCD = necrotic + AL-PCD) were plotted. Error bars = standard error of n = 3 replicates.

When *Arabidopsis thaliana *cell suspension cultures are subjected to a range of temperatures the percentage of cells undergoing AL-PCD increases as the temperature increases, with the highest rate of AL-PCD being observed around 55°C [11,2]. However, when cells are subjected to a heat treatment above 55°C, the rate of AL-PCD starts to decrease and the rate of necrosis increases (Figure 3A). The fate of a cell undergoing a certain stress can be thought of as having two thresholds: the point at which the stress induces the cell to undergo PCD (alive/PCD threshold), and the point at which the cell is no longer able to control its fate and undergoes uncontrolled cell death (PCD/necrosis threshold) [8]. A curve can be plotted to show the response to heat stress over a range of temperatures and this is invaluable when investigating whether a certain compound/gene can modulate the cells PCD response, whether this be a suppression of PCD or shift in alive/PCD or PCD/necrosis thresholds. We investigated, therefore, whether the root hairs showed a similar temperature dependant response. Figure 3A shows the AL-PCD response curve achieved when 7 day old *Arabidopsis thaliana *cells are subjected to increasing temperatures for ten minutes and scored for AL-PCD morphology. A similar AL-PCD response curve was observed in root hairs of 5 day old seedlings subjected to the same stress treatment (Figure 3B). The percentage of AL-PCD root hairs increases with the temperature increase up to 65°C, whereas further increases of temperature cause root hairs to die by necrosis rather than AL-PCD.

### Response of root hairs to ethanol

**Figure 4:**
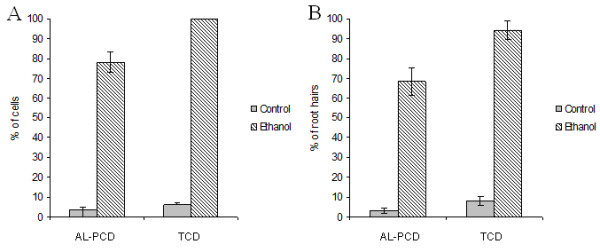
**The effect of ethanol on rates of AL-PCD in *Arabidopsis thaliana***. Seven day old cell suspension cultures **(A) **and 5 day old seedlings **(B) **of *Arabidopsis thaliana *were treated with 10% ethanol and then scored for AL-PCD 24 hr (cells) or 6 hr (root hairs) later. Cells/root hairs showing retraction and condensation of the cytoplasm and no FDA staining were deemed to have undergone AL-PCD. Those root hairs/cells showing neither FDA staining nor retraction of the cytoplasm were scored as necrotic. Rates of AL-PCD or Total Cell Death (TCD) (TCD = Necrotic + AL-PCD) were plotted. Error bars = standard error of n = 3 replicates.

Previous experiments in our laboratory have shown that ethanol is an effective inducer of AL-PCD in cell cultures [6] and provides an alternative to heat stress, which may not be always be a suitable inducer of death for certain investigations. Therefore a comparison was made of the ability of ethanol to induce AL-PCD in both cell culture and root hair systems. Figure 4 shows the levels of AL-PCD induced in cultured cells (Figure 4A) and root hair cells (Figure 4B) following an incubation with 10% ethanol and scoring for AL-PCD morphology either after 24 hr (cells) or 6 hr (root hairs). In both root hair cells and suspension culture cells, ethanol induces significant levels of AL-PCD (78% AL-PCD; 100% TCD (total cell death = necrotic + AL-PCD) in cell cultures and 68% AL-PCD; 94% TCD in root hairs). This indicates that not only can ethanol be used as an alternative method to induce AL-PCD, but that root hairs undergo AL-PCD in response to a variety of stresses.

### Response of root hairs to salt stress (NaCl), acetylsalicylic acid (ASA) and hydrogen peroxide

**Figure 5:**
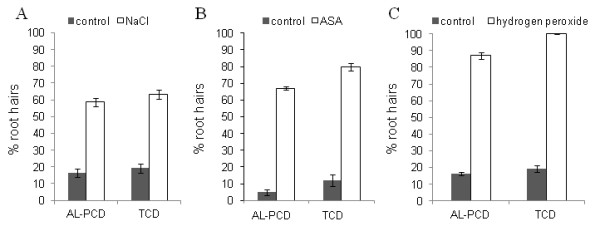
**The effect of sodium chloride, ASA and hydrogen peroxide on rates of AL-PCD in *Arabidopsis thaliana *root hairs**. Five day old seedlings of *Arabidopsis thaliana *were treated subjected to treatment with NaCl, ASA and H_2_O_2 _and then scored for AL-PCD 24 hr later. Root hairs showing retraction and condensation of the cytoplasm and no FDA staining were deemed to have undergone AL-PCD. Those root hairs showing neither FDA staining nor retraction of the cytoplasm, were scored as necrotic. Rates of AL-PCD or Total Cell Death (TCD) (TCD = necrotic + AL-PCD) were plotted. Error bars = standard error of n = 3 replicates.

It has been shown that salicylic acid plays a role in plant defence responses and recently it was shown that acetylsalicylic acid (ASA) could induce PCD in *Arabidopsis thaliana *cell cultures [28]. ASA also induces apoptosis in animal cells, e.g. in human colon cancer cell line SW480 [29]. In plants hydrogen peroxide is generated in response to a wide range of abiotic and biotic stresses and has been shown to induce PCD in cells [15-17]. Salt stress is known to induce PCD in plants and has recently been reviewed by Shabala [30]. NaCl, ASA and hydrogen peroxide therefore, provide three biologically relevant inducers of PCD in plants. As such the ability of NaCl, ASA and hydrogen peroxide to induce AL-PCD in root hairs was investigated. All three compounds tested were able to induce AL-PCD in roots hairs (Figure 5). Figure 5 shows that pre-incubation for 5 min with 100 mM NaCl (Figure 5A) or incubation for 24 hrs with 0.08 mM ASA (Figure 5B) led to 58% and 67% of root hairs displaying the AL-PCD morphology respectively as opposed to 16% and 5% for the control root hairs. After 24 hr incubation in 25 mM H_2_O_2 _it can be seen that hydrogen peroxide induced AL-PCD in root hairs with 87% of root hairs displaying the AL-PCD morphology as a opposed to 16% in control root hairs (Figure 5C). These results show that this novel root hair assay can be used as an *in vivo *system to quantify alterations in rates of AL-PCD in response to biological inducers of PCD in plants.

### Modulation of AL-PCD levels in the At-DAD1 over-expressing line

**Figure 6:**
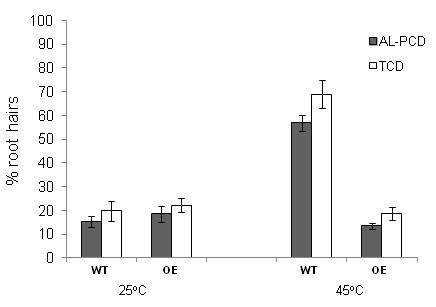
**AL-PCD rates in the *DAD1 *over-expressing line of *Arabidopsis thaliana***. Five day old seedlings of *DAD1 *over-expressing (OE) and wild-type (WT) lines of *Arabidopsis thaliana *were subjected to a heat treatment at 45°C for 10 min or no heat treatment. Root hairs were scored 24 hr later and root hairs showing retraction and condensation of the cytoplasm and no FDA staining were deemed to have undergone AL-PCD. Root hairs showing neither FDA staining nor retraction of the cytoplasm, were scored as necrotic. Rates of AL-PCD or Total Cell Death (TCD) (TCD = necrotic + AL-PCD) were plotted. Error bars = standard error of n = 3 replicates.

With the undertaking of many large scale mutagenesis programmes, and advances in plant transformation, there are many avenues open for the investigation of PCD and the genes involved in the PCD process in plants. It is not feasible however, to establish cell suspension cultures for each mutant or transgenic line in order to investigate PCD. A major advantage in using this *in vivo *root hair system would be to evaluate rates of AL-PCD in mutant/transgenic plant lines without the need to establish cell suspension cultures. DAD-1 is an ER localised protein [27]. When mutant hamster cells lines, that undergo apoptosis under restrictive temperature, are transfected with an expression vector containing *At-DAD1*, At-DAD1 can successfully rescue these cells and promote survival [26,31]. In addition, transient expression of At-DAD1 and At-DAD2 (a homologue of *At-DAD1*) were able to suppress the onset of ultraviolet C induced DNA fragmentation in protoplasts of *Arabidopsis thaliana *[27]. The transient expression studies carried out by Danon *et al*. [27] suggest that over-expression of At-DAD1 might be able to modulate levels of AL-PCD in plants. In order to test this, and to determine the usefulness of this method for evaluating rates of PCD in mutant/transgenic plant lines, we investigated levels of AL-PCD in lines over-expressing At-DAD-1. Figure 6 shows that when no stress is applied to the root hairs the line over-expressing At-DAD1 shows similar background levels of AL-PCD to the wild-type line. This demonstrates that under non-stressed conditions root hairs in these lines do not show alteration of their basal AL-PCD rates. Seedlings were subjected to heat treatment in order to ascertain if altering DAD-1 expression had any effect on rates of AL-PCD. We have previously used heat treatment to report on the effect of peroxidase gene expression in tobacco cell cultures [18]. In this study it was found that 45°C for 10 minutes, which is at the alive/PCD threshold, was the optimal temperature for reporting on changes in rate of AL-PCD between transgenics and wild-type cultures. When roots of wild-type and transgenic *Arabidopsis thaliana *are subjected to a heat stress of 45°C there is a marked difference between the levels observed in the wild-type when compared to the levels seen in the DAD-1 over-expressing line. Over-expression of DAD-1 reduces AL-PCD to levels observed when no stress is applied (Figure 6). Whether it is a complete suppression of AL-PCD or merely a shift in the threshold of AL-PCD induction requires further investigation. It may be that the over-expression of At-DAD1 increases the levels of a "pro-survival" signal which affords some cellular protection. This experiment does demonstrate that the DAD-1 protein has the ability to modulate AL-PCD rates in *Arabidopsis thaliana in vivo*, and that the root hair model can be used to investigate changes in levels of AL-PCD when the expression level of a specific gene is altered. In the future, this *in vivo *root hair PCD assay will facilitate identification of gene modifications in mutants/transgenic lines that have a role in regulating cell death responses in plant cells.

### AL-PCD in other plant systems

**Figure 7:**
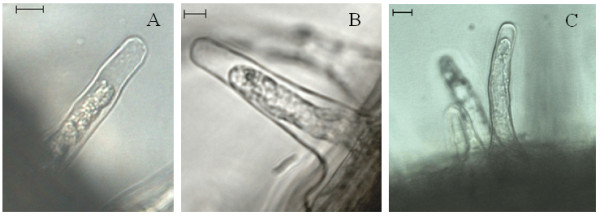
**AL-PCD morphology in root hairs or *Medicago truncatula, Zea mays *and *Quercus robur***. **(A) **Root hair of *Medicago truncatula *24 hr after a 10 min heat shock at 55°C; **(B) **Root hair of *Zea mays *6 hr after a 10 min heat shock at 55°C; **(C) **Root hair of *Quercus robur *6 hr after a 10 min heat shock at 55°C. In all three species **(A-C) **retraction and condensation of the cytoplasm is observed and root hairs stained negative for FDA indicating the root hairs had undergone AL-PCD. Scalebar: 10 μm.

While *Arabidopsis thaliana *is a model plant for examining many plant processes, in certain cases it is more appropriate to investigate regulation of PCD in other species. We investigated whether the AL-PCD morphology observed in *Arabidopsis thaliana *could be seen in *Medicago truncatula, Zea mays*, or *Quercus robur*. Figure 7 shows that in root hairs of all three species subjected to a 10 minute heat stress at 55°C, a retraction of the cytoplasm away from the cell wall and condensation of the cytoplasm was observed. The morphology in most cases was comparable to that which is observed when root hairs of *Arabidopsis thaliana *are subject to heat stress. In certain root hairs, however, the cytoplasm did not retract as a complete unit and occasionally a splitting of the cytoplasm into two or more parts was observed. When this occurred, fragments of the cytoplasm were left behind at the root hair tip as the cytoplasm retracted towards the base of the root hair (data not shown).

The applicability of the this novel *in vivo *root hair technique to plants other than *Arabidopsis thaliana *was confirmed by the induction of AL-PCD morphology in root hair cells of *Medicago truncatula, Zea mays *and *Quercus robur *and provides an effective model for the investigation of AL-PCD in, we believe, any plant species. It will facilitate the investigation of PCD in the adaptation, or lack thereof, of crop responses to abiotic stress such as cold, drought and salinity. For example, it could be envisaged that the induction of AL-PCD in root hairs of *Zea mays *undergoing cold stress may be an indicator of cold susceptibility/tolerance and provide a rapid initial screen for breeding lines of *Zea mays *that are adapted to a more temperate climate. The assessment of AL-PCD in root hairs can easily be conducted at different stages of plant growth and development. It is possible certain species have a greater tolerance to different abiotic stresses and the threshold for the induction AL-PCD by heat stress may be at a lower or higher temperature than that seen in this study. Our investigations to date suggest the AL-PCD morphology is a feature of most, if not all, plant species and can therefore be used to investigate regulation of AL-PCD in plants.

## Conclusions

We describe a rapid and adaptable *in vivo *root hair model for accurately assessing rates of AL-PCD in plants. We show that, as in cell suspension cultures, abiotic stress (heat) or chemical stress (ethanol, ASA, NaCl, H_2_O_2_) induce cell death in root hairs that results in the AL-PCD corpse morphology. This morphology is associated with caspase-like activity. Using an At-DAD1 over-expressing line we demonstrate that this system can be used for screening lines altered in the expression of genes which are thought to be involved in PCD for perturbations in rates of AL-PCD. The transferability of this system to other plants, including an important crop species (*Zea mays*), is also demonstrated.

## Methods

### Cell suspension cultures growth

Cell suspension cultures of *Arabidopsis thaliana *were grown essentially as described in [32]. Briefly cultures were grown in 100 ml of liquid Murashige and Skoog (basal salts 4.3 g l^-1 ^) medium containing 0.5 mg l^-1 ^NAA, 0.05 mg l^-1 ^kinetin and 3% (w/v) sucrose, pH 5.8 with agitation on an orbital shaker (100 rev/min) under low light conditions (6 μmol m^-2 ^s^-1 ^) in a controlled environment room, 23°C, 24 hr light. Cultures were subcultured every 7 days by taking 10 ml and adding to 100 ml of fresh medium.

### Heat treatment of cell suspension cultures

Ten ml of seven day old cultures was placed in sterile 100 ml flasks. The flasks were then placed in a shaking (85 oscillations/minute) waterbath (Grant OLS200) set to the desired temperature for ten minutes. Following heat treatment flasks were returned to the controlled environment room, with shaking, until scoring 24 hr later.

### Plant growth

Seeds of *Arabidopsis thaliana, Medicago truncatula *and *Zea mays *were sterilised in 20% (V/V) commercial bleach (final concentration of NaOCl approximately 1%) followed by washing four times with sterile distilled water (SDW). Seeds of *Medicago truncatula *were scarified using fine sandpaper before sterilisation for 3 min in 20% (V/V) commercial bleach. *Zea mays *and *Arabidopsis thaliana *seeds where sterilized for twenty minutes. Following sterilisation all seeds where plated in a single line on their respective growing medium to allow the roots to grow down the surface. *Arabidopsis thaliana *seeds were plated on semi-solid half-strength Murashige and Skoog (basal salts, 2.15 g l^-1 ^) medium, 1% sucrose and 1.5% agar in 9 cm Petri dishes and stratified at 4°C for 24 hr, before being placed vertically at 22°C, 16 hr light, 8 hr dark. *Zea mays *seeds were germinated on Whatman filter paper No.1 that had been soaked in half-strength Hoagland's medium (1.6 g l^-1 ^, Sigma) in square Petri dishes (12 cm × 12 cm), five seeds per Petri dish. The Petri dishes where then sealed with parafilm wrapped in aluminium foil and placed vertically at 22°C. Following sterilisation seeds of *Medicago truncatula *were left to imbibe for 24 hr at 4°C and then plated on nitrogen-free modified Fahraeus medium [33] (1 mM CaCl_2_, 0.5 mM MgSO_4_, 0.7 mM KH_2_PO_4_, 0.8 mM Na_2_HPO_4_, 50 μM FeEDTA (pH 6.5) including the following microelements, CuSO_4_, MnSO_4_, ZnSO_4_, H_3_BO_3_, and Na_2_MoO_4 _at a final concentration of 0.1 mg l^-1 ^) with 1.5% w/v agar in square (12 cm × 12 cm) Petri dishes, six seeds per plate. Following three days at 4°C the plates were placed vertically at 24°C day (16 hr), 18°C night (8 hr).

For the germination of *Quercus robur*, acorns were soaked at 4°C for 5 days in water. Following soaking acorns were half buried in vermiculite and covered with damp tissue. Acorns were then placed at 22°C, 16 hr light, 8 hr dark, and watered generously every second day. Three to four weeks later seedlings were selected where significant root growth had occurred. Following removal of the vermiculite from the roots by gentle washing in SDW, seedlings were then subjected to the heat treatment as described below.

### Heat treatment of root hairs

All heat treatments were carried out in SDW using a Grant OLS200 waterbath set the desired temperature, without shaking, for ten minutes. Five day old seedlings of *Arabidopsis thaliana *were carefully transferred with the forceps from the growth medium to 15 ml Falcon tubes containing 10 ml of sterile distilled water (SDW). Seed germination on the vertically positioned Petri dishes prevented developing roots from penetrating the surface of the medium and therefore reduced the background root hair damage caused by transfer from the growth plate. Following heat treatment seedlings were then returned to the constant temperature room at 23°C in the light for 6 hr, after which AL-PCD was scored. Both *Medicago truncatula *and *Zea mays *were heat treated in 6 cm Petri dishes containing 5 ml of SDW. Five day old seedlings were used for heat treatment *Medicago truncatula *and 3 day old seedlings were used for *Zea mays*. Following heat treatment, seedlings of *Medicago truncatula *were returned to the controlled environment room 24°C day (16 hr), 18°C night (8 hr) and scored for AL-PCD 24 hr later. Seedlings of *Zea mays *were incubated in the dark at room temperature following heat treatment for 6 hr until scored for AL-PCD. Three to four week old *Quercus robur *seedlings were heat treated in square Petri dishes (10 cm × 10 cm) filled with approximately 50 ml of SDW. Following heat treatment, seedlings were incubated in the light at room temperature for 6 hr before scoring for AL-PCD.

### Ethanol treatment

Ethanol (Merck) was added to 7 day old cells at a final concentration of 10% (v/v). Cells were then returned to the controlled environment room with shaking (100 rev/min) for 24 hr until scoring. Five day old *Arabidopsis thaliana *seedlings were placed in 6 cm Petri dishes containing 5 ml of 10% ethanol (v/v) (Merck). Petri dishes containing the seedlings were then incubated at room temperature for 6 hr until scoring.

### ASA treatment

Five day old *Arabidopsis thaliana *seedlings were placed in 6 cm Petri dishes containing 5 ml of 0.08 mM ASA solution (Sigma). The Petri dishes containing the seedlings were then incubated at room temperature in constant light for 24 hr until scoring.

### NaCl treatment

Five day old *Arabidopsis thaliana *seedlings were incubated in 6 cm Petri dishes containing 5 ml of 100 mM NaCl solution (Sigma) for 5 min. Afterwards seedlings were transferred to 6 cm Petri dishes containing 5 ml of SDW and kept in constant light at room temperature for 24 hr until scoring.

### H_2_O_2 _treatment

Five day old *Arabidopsis thaliana *seedlings were incubated in 6 cm Petri dishes containing 5 ml of 25 mM H_2_O_2 _solution (Sigma) for 5 min. The Petri dishes containing the seedlings were then incubated at room temperature in constant light for 24 hr until scoring.

### Caspase inhibitor study

Prior to heat-treatment, five day old *Arabidopsis thaliana *seedlings were incubated for 1 hour in 6 cm Petri dishes containing 5 ml of 1 μM Ac-DEVD-CHO (A.G. Scientific) in SDW. Subsequently Petri dishes were transferred to a waterbath set to 49°C for ten minutes. Following heat treatment seedlings were incubated at room temperature in constant light for 24 hr until scoring. Ac-DEVD-CHO stock was prepared in DMSO (10 mg/ml) and used within one month. DMSO solvent control had no influence on root hairs viability (data not shown).

### AL-PCD assay

Cells/roots were stained with fluorescein diacetate (FDA) a live/dead stain. Only viable cells/root hairs are able to cleave FDA to form fluorescein which, when excited by a wavelength of 485 nm, fluoresces green. Root hairs/cells were stained in a 1 μg/ml solution of FDA on the standard microscope slides and immediately examined under white light and fluorescent light. Whole Arabidopsis seedlings were placed on the slides; whereas only roots of *Medicago trunculata, Zea mays *and *Quercus robur *were used to facilitate placing the cover slip on the top of the specimen. As root hairs are single cells on the root surface, the morphology of individual root hairs along the main root can be readily observed. Root hairs/cells that were positive for FDA staining were scored as alive. Root hairs/cells negative for FDA staining were examined further and scored as either AL-PCD, having a retracted cytoplasm, or necrotic, having no retracted cytoplasm and therefore no distinguishable morphology compared to living cells under the light microscope. The percentage for each category was calculated as a percentage of the total number of roots hairs scored (typically ~ 100) averaged over at least three replicates. Images were taken using either an Olympus BX60 or Leica DML8 microscope with Leica DFC240C colour camera attached and captured using Leica application suite V3.1.0 software. Data is expressed in graphs as AL-PCD and Total Cell Death (TCD, where TCD = AL-PCD + necrosis).

## Competing interests

The authors declare that they have no competing interests.

## Authors' contributions

EMM initially designed the assay and contributed the results for *Arabidopsis thaliana *and the DAD-1 mutant. BVH and JK optimised the design of the assay. BVH performed the AL-PCD assay on *Zea mays *and *Medicago trunculata*, JK performed the assay on *Quercus robur*. TG, COR and PMcC sourced funding for the project. PG suggested and provided the DAD1 transgenics. PMcC conceived original hypothesis and oversaw project. BVH drafted the initial version of the manuscript and all authors contributed to subsequent manuscript preparation and approved the final version.
